# Influence of mouse strain on ovarian tissue recovery after engraftment with angiogenic factor

**DOI:** 10.1186/s13048-015-0142-6

**Published:** 2015-03-27

**Authors:** Maïté Fransolet, Laurie Henry, Soraya Labied, Marie-Caroline Masereel, Silvia Blacher, Agnès Noël, Jean-Michel Foidart, Michelle Nisolle, Carine Munaut

**Affiliations:** Laboratory of Tumor and Developmental Biology, GIGA-R, University of Liège, Tour de Pathologie (B23), Sart Tilman, B-4000 Liège, Belgium; Department of Obstetrics and Gynecology, University of Liège, Hôpital de la Citadelle, B-4000 Liège, Belgium

## Abstract

**Background:**

For women facing gonadotoxic treatment, cryopreservation of ovarian tissue with subsequent retransplantation during remission is a promising technique for fertility preservation. However, follicle loss within grafted ovarian tissue can be caused by ischemia and progressive revascularization. Several xenograft models using different immunodeficient rodent lines are suitable for studying ovarian tissue survival and follicular viability after frozen-thawed ovarian cortex transplantation. SCID mice, which are deficient for functional B and T cells, are the most commonly used mice for ovarian xenograft studies. However, due to incomplete immunosuppression, NOD-SCID mice displaying low NK cell function and an absence of circulating complement might be more appropriate. The present study aims to define the most appropriate immunodeficient mouse strain for ovarian tissue xenotransplantation by comparing ovarian graft recovery in SCID and NOD-SCID mice following engraftment in the presence of isoform 111 of vascular endothelial growth factor.

**Methods:**

Sheep ovarian cortex fragments were embedded in a collagen matrix, with or without VEGF_111_, before being stitched onto the ovaries of SCID and NOD-SCID mice. Transplants were recovered after 3 days to study early revascularization or after 3 weeks to evaluate follicle preservation and tissue fibrosis through histological analyses.

**Results:**

At day 3, vessels were largely reorganized in the ovarian grafts of both mouse strains. After 3 weeks, the cortical tissue was clearly identifiable in SCID mice but not in NOD-SCID mice. Upon VEGF_111_ treatment, vascularization was significantly improved 3 days after transplantation in SCID mice. This increase in vessel density was correlated with better follicular preservation in SCID mice 3 weeks after transplantation. Fibrosis was not decreased by VEGF treatment in either mouse strain.

**Conclusions:**

Tissue architecture and follicular morphology were better preserved in ovarian tissues grafted in SCID mice in comparison with NOD-SCID mice. Moreover, tissue revascularization was improved in SCID mice by VEGF_111_ graft treatment. Thus, we consider SCID mice to be the best murine model for studying ovarian tissue xenografts.

## Background

To date, the future fertility of women with cancer remains a major concern because these women often face ovarian damage and premature ovarian failure after gonadotoxic treatments. For patients who need immediate chemotherapy or for prepubertal girls, ovarian cortex cryopreservation represents a promising fertility preservation technique [1]. Thus far, autologous transplantation of frozen/thawed ovarian tissue has allowed for the birth of more than 30 babies worldwide [2].

For autologous transplantation of frozen/thawed ovarian tissue, graft neovascularization remains a limiting factor for survival. Indeed, the lack of vascular anastomosis after tissue transplantation is responsible for early transplant ischemia, which decreases the ovarian tissue survival rate and gives rise to massive follicular loss by apoptosis [3-7]. To study ovarian tissue survival and follicular viability after frozen-thawed ovarian cortex transplantation, several xenograft models using different immunodeficient rodent lines have been developed and published. Indeed, *in vivo* research using preclinical models represent a crucial step before clinical application. Severe combined immunodeficient (SCID) mice were the first and most commonly used mice for ovarian xenografts [5,8,9]. This mouse strain is characterized by the *scid* mutation, which leads to a defect in the recombination of antigen receptor genes, impairing the capacity to generate functional B and T lymphocytes [10,11]. This deficiency contributes to the acceptance of xenogeneic grafts without severe rejection. However, in 2 to 25% of young adult mice, the mutation is not completely stable [12]. In this context, development and maturation of lymphocytes can occur and lead to incomplete immunosuppression, which may limit the grafting efficiency of xenogeneic tissues [13]. Therefore, long term foreign tissue rejection represents an important issue in SCID mice. A few years after SCID mice generation, the *scid* mutation was transferred into a Non-Obese Diabetic (NOD) background. This transfer leads to NOD-SCID mice, which have reduced NK cell activity in addition to the deficiency in functional B and T cells [14]. Moreover, their ability to activate some components of the complement system is impaired, and these mice are markedly deficient in macrophages [15]. Based on these multiple defects in innate and adaptive immunity, the NOD-SCID strain is expected to be more promising as a tool for xenotransplantation.

To reduce the hypoxic period after tissue transplantation and improve follicular preservation, angiogenesis can be stimulated by vascular endothelial growth factor, which is the main signaling protein that regulates new vessel formation from pre-existing vessels. The effects of two VEGF-A isoforms, VEGF_111_ and VEGF_165_, were recently tested in a xenograft model developed in our lab using SCID mice [16,17]. These VEGF isoforms were chosen for their unique properties; VEGF_111_ is soluble and resistant to proteolysis, whereas VEGF_165_ is additionally anchored to the extracellular matrix. Both VEGF isoforms increase blood vessel recruitment and functional angiogenesis.

Due to differences in immune response, the mouse strain used in the xenograft model can significantly impact the results. The aim of the present study was to determine the most appropriate experimental model, SCID or NOD-SCID mice, for ovarian cortex transplantation. Histological and functional features of frozen-thawed sheep ovarian cortex with or without VEGF_111_ treatment were subsequently analyzed.

## Methods

### Collection and preparation of ovarian tissue

Ovarian cortex from sheep was obtained and prepared as described previously [16]. The Animal Ethics Committee of the University of Liège approved the use of sheep ovarian tissue obtained from the Ovine Research Center (University of Namur). Briefly, after euthanasia, ovaries were transferred and kept at 4°C until processing in Leibovitz L-15 medium (Lonza, Verviers, Belgium, BE12-700 F) supplemented with 10% normal sheep serum (Hormonology Laboratory, Marloie, Belgium). In the laboratory, the medulla was removed and the cortex was cut into strips (5 × 5 × 1 mm) before equilibration during 30 min at 4°C in cryopreservative medium containing Leibovitz L-15 medium supplemented with 10% normal sheep serum, 10% dimethylsulfoxide (1.5 M) (Sigma-Aldrich, Bornem, Belgium) and 0.1 M sucrose.

### Freezing and thawing of ovarian tissue

Ovarian biopsies were cooled in a programmable freezer (CL-8800i system, CryoLogic, Mulgrave, Victoria, Australia) in cryovial tubes (Simport, Montreal, Quebec, Canada) as described previously [18] and stored in liquid nitrogen until transplantation. At transplantation, the cryovials were incubated at room temperature for 2 min and thawed by rapid immersion at 37°C in a water bath. To remove the cryoprotectant, cortical fragments were washed three times for 5 min at 37°C in serum-free Leibovitz medium.

### Ovarian transplant encapsulation

Cortical biopsies of 2.5 × 2.5 × 1 mm from 20 ewes were embedded in a three-dimensional collagen matrix as previously described [16]. On a heating plate, a first layer of type I collagen extracted from the tail tendons of rats [19] was poured in an agar ring. The collagen matrix was prepared by mixing 9 volumes of type I collagen (2.4 mg/ml), 1 volume of 10× minimal essential medium and approximately 0.1 volume of 1 M NaOH to adjust the pH to 7.4. After polymerization, frozen-thawed ovarian strips pierced with surgical thread were placed and overlaid by a second layer of type I collagen.

VEGF_111_ was produced and purified as previously described [16,20] and then included in the collagen of treated group at a concentration of 100 nM.

### Mouse transplantation and sacrifice

Eight-week-old severe combined immunodeficient (SCID, n = 32) or non-obese diabetic SCID (NOD-SCID, n = 19) mice (Charles River, Saint-Germain-sur-l’Arbresle, France) were anesthetized with an intraperitoneal injection of ketamine (100 mg/kg) and xylazine (10 mg/kg). An encapsulated ovarian biopsy was stitched onto the ovary with a 7–0 Prolene suture. Fragments from 2 different ewes were usually used per experiment.

To study the functional vascular network, 200 μl of dextran/fluorescein isothiocyanate (FITC) (2.5 mg/ml in PBS) (Sigma-Aldrich, Belgium) was intravenously injected 3 min before sacrifice. Animals were euthanized by cervical dislocation 3 days or 3 weeks after grafting. The sheep ovarian transplants were recovered and fixed in 4% formaldehyde.

### Histological assessment

Each graft was cut into 5-μm-thick serial sections. Ten sections per transplant, which covered the entire ovarian fragment, were stained with haematoxylin and eosin (H&E).

To detect the functional vascular network, immunostaining was performed in one step. Sections were deparaffinized and rehydrated, and endogenous peroxidase activity was blocked by incubating the sections in 3% hydrogen peroxide for 20 min at room temperature (RT). Non-specific binding sites were blocked by incubation with phosphate-buffered saline (PBS) containing 10% bovine serum albumin for 30 min at RT. The sections were incubated with a ready-to-use anti-fluorescein antibody (Converter-POD, Roche, France), which directly recognized the FITC molecules fixed on dextran, for 30 min at RT. Negative control slides were also performed by replacing the antibody with phosphate-buffered saline (PBS). The 3,3′-diaminobenzidine substrate (K3468, Dako, Belgium) allowed the visualization of positively stained cells, and the sections were counterstained with haematoxylin.

Fibrosis was evaluated using saffron staining. Sections were incubated in 1% saffron solution (Microm Microtech, Francheville, France) in 100% ethanol for 10 min at 37°C.

### Virtual image acquisition

Slides were digitized in brightfield at 20× magnification using the NanoZoomer 2.0-HT C9600-13 (Hamamatsu Photonics, K.K., Japan), a high-speed and high-resolution digital slide scanner system. Digital images were acquired in .ndpi format and visualized with NDP.view software (Olympus) on a standard PC.

### Histological analysis of follicle numbers

At least 10 H&E sections per fragment were analyzed by Image J software in order to limit the impact of natural follicular heterogeneity on experimental results [21]. Follicles were quantified manually and, to avoid double counting, only follicles with a visible nucleus were taken into account. Follicles were then classified according to their maturity (primordial, primary or secondary follicles). Follicular densities (number/mm^2^) were calculated after a manual surrounding of the cortical surface (Image J software).

### Vascular network analysis

Stained vessels were drawn manually on digital images and transformed to obtain a binarized image in which the intensity of pixels representing vessels was set to 1 and that corresponding to the background was set to 0. The number of vessel sections was measured automatically on the binary images [22,23]. The results are expressed as unity of area of ovarian tissue. An image analysis was conducted using MATLAB 8.1.0.604 (R2013a) software (Mathworks, Inc.).

### Fibrosis analysis

Digital images of saffron-stained tissue slices were analyzed using ImageJ software (National Institutes of Health, USA). Digitized color images were first decomposed into their red, green, and blue components. To enhance the features of fibrosis, the blue component was subtracted from the red component. The resulting images were then binarized using an appropriate threshold. In this method, pixels that were associated with fibrosis were assigned a value of 1, and pixels that were associated with non-fibrotic tissue were assigned a value of 0. The ratio of the surface area that was occupied by fibrotic tissue to the total surface area of the tissue represented the proportion of fibrotic tissue in each transplant.

### Statistical analysis

Statistical analyses were performed using GraphPad Prism software (GraphPad, San Diego, CA). The Mann–Whitney test was applied for comparisons between two different groups. The proportion of positive fragments for dextran/FITC-stained blood vessels was compared with a chi-square test. A probability of *p* < 0.05 was considered to be statistically significant.

## Results

### Graft recovery and histological examination

**Figure 1:**
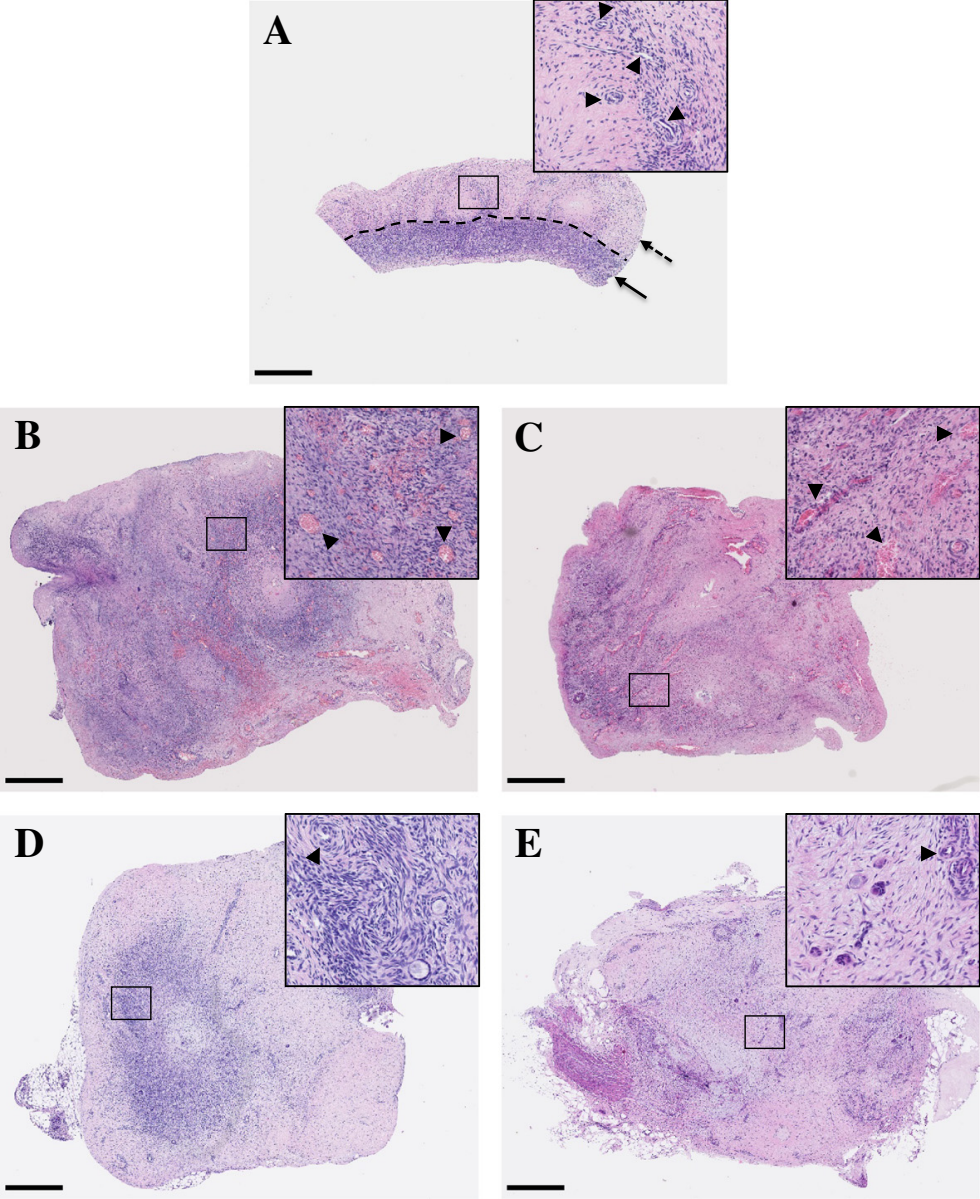
**Histological analysis of haematoxylin and eosin sections of ovarian tissue.** Representative illustrations of fresh ovarian tissue before cryopreservation (the cortex and medulla are identified by a plain and dotted arrow, respectively) **(A)**; ovarian control grafts 3 days after transplantation in SCID mice **(B)** and NOD-SCID mice **(C)**; ovarian control grafts 3 weeks after transplantation in SCID mice **(D)** and NOD-SCID mice **(E)**. ► Indicates vessels. Scale bar: 500 μM. Images are representative of at least 2 experiments.

Mice were sacrificed 3 days or 3 weeks after transplantation, and all 102 ovarian xenografts were recovered from the SCID (n = 32) and NOD-SCID (n = 19) mice. For technical reasons, two transplants could not be analyzed. The cortex and medulla were clearly detectable on fresh ovarian tissue. Most of the follicles were located in the cortex, whereas the medulla contained a highly vascular stroma (Figure 1A). After grafting, haematoxylin and eosin staining of tissue sections revealed major remodeling of the preexisting vessels compared to fresh tissue as soon as 3 days after transplantation in both mouse strains (Figure 1B, C). Nevertheless, at day 3 post transplantation, the tissue architecture, cortex and medulla were still recognizable. In sharp contrast, grafts recovered after 3 weeks of transplantation displayed extensive tissue remodeling in both mouse strains (Figure 1D, E). The overall tissue histology displayed better morphology in SCID compared to NOD-SCID mice. The cortex of the biopsy transplanted in SCID mice was easily identified, while the tissue grafted in NOD-SCID mice appeared more disorganized.

No effect of VEGF_111_ on ovarian tissue fragment histology was observed at 3 days or 3 weeks post transplantation.

### Graft neovascularization

**Figure 2:**
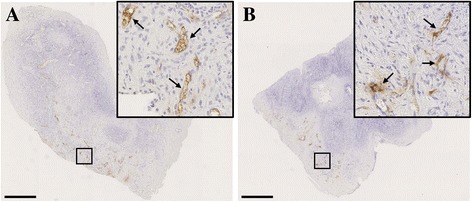
**Functional vascular network identification in ovarian tissue 3 days after transplantation in SCID and NOD-SCID mice.** Representative illustrations of functional blood vessels (identified by plain arrows) revealed by dextran-FITC immunostaining in VEGF_111_ treated fragments grafted in SCID **(A)** and NOD-SCID **(B)** mice. Scale bar: 500 μM. Images are representative of at least 2 experiments.

**Figure 3:**
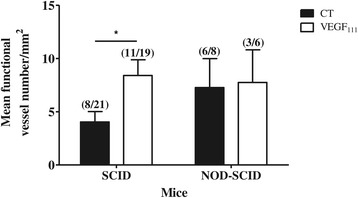
**Functional vascular network analysis of ovarian tissue 3 days after transplantation in SCID and NOD-SCID mice.** Quantification of functional vessel density in ovarian transplants with or without VEGF_111_ treatment grafted in SCID or NOD-SCID mice. *p < 0.05.

Dextran/FITC injections were performed before sacrifice, and perfused transplants were evaluated at day 3 post transplantation by immunohistochemical detection of FITC (Figure 2). Treatment with VEGF_111_ improved the percentage of perfused transplants only in SCID mice (Table 1). Functional blood vessel density was also higher in fragments grafted in SCID mice following treatment with VEGF_111_ (Figure 3).

**Table 1:** **Percentage of ovarian transplants positive for dextran/FITC staining 3 days after transplantation in SCID and NOD-SCID mice**

	**n (%)**	**n (%)**
**SCID**	Negative	Positive
CT (n = 21)	13 (62)	8 (38)
VEGF_111_ (n = 19)	8 (42)	11 (58)
**NOD-SCID**	Negative	Positive
CT (n = 8)	2 (25)	6 (75)
VEGF_111_ (n = 6)	3 (50)	3 (50)

### Follicular morphology and density

**Figure 4:**
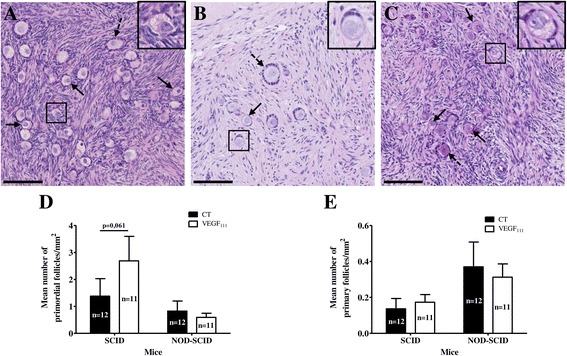
**Follicle analysis of ovarian tissue 3 weeks after transplantation in SCID and NOD-SCID mice.** H&E sections of fresh ovarian tissue **(A)** and ovarian control tissue transplanted in SCID **(B)** and NOD-SCID **(C)** mice (primordial and primary follicles are identified by plain and dotted arrows, respectively). Quantification of primordial **(D)** and primary **(E)** follicle density in ovarian transplants with or without VEGF_111_ treatment and grafted in SCID or NOD-SCID mice. Scale bar: 100 μM. Images are representative of at least 2 experiments.

Morphologically, follicles from tissue grafted for 3 weeks in both mouse strains were indistinguishable from those from fresh tissue (Figure 4A). Follicles from SCID mouse transplanted tissue were round and contained fewer vacuoles (Figure 4B). In NOD-SCID mice, despite a well-organized granulosa cell layer and intact oocytes, follicles displayed ooplasm with many vacuoles (Figure 4C).

To determine if ovarian cortex treatment with VEGF_111_ could improve follicular survival, follicular density was evaluated in SCID and NOD-SCID mice (Figure 4D, E). Compared to vehicle treated controls, VEGF_111_ had an impact on primordial follicle preservation in SCID mice.

### Fibrosis analysis

**Figure 5:**
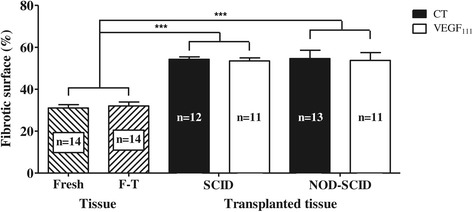
**Fibrosis analysis of ovarian tissue 3 weeks after transplantation in SCID and NOD-SCID mice.** Percentage of fibrotic tissue in fresh and frozen-thawed (F-T) ovarian biopsies and ovarian transplants grafted in SCID and NOD-SCID mice. ***p < 0.001.

To further the tissue remodeling analysis, fibrosis was evaluated by saffron staining. Analysis of fresh and frozen-thawed tissues compared to tissue grafted over 3 weeks showed that fibrosis was significantly increased after transplantation. Among grafted ovarian tissues, fibrotic areas were found to be similar in controls and in VEGF-treated groups, regardless of the mouse strain (Figure 5).

## Discussion

Fertility restoration after transplantation of frozen-thawed ovarian tissue is a major concern in reproductive medicine. To improve the process, many xenograft models have been used [5]. Indeed, establishing rapid perfusion of the transplant associated with a significant follicular preservation in a mouse model could be very useful in the clinic for human ovarian tissue autotransplantation. This study was undertaken to compare results obtained after ovarian tissue xenotransplantation in two different immunodeficient mice strains. Using a pre-clinical mouse model, we provide evidence that the morphological features of transplanted ovarian tissue could be impacted by the mouse strain. Our results indicate that graft perfusion and follicular preservation were better in ovarian fragments grafted in SCID mice.

The period before revascularization of frozen-thawed ovarian tissue grafts appears to be the main factor responsible for the ischemic damage that decreases the lifespan of the transplant [3,24]. The electron paramagnetic resonance oximetry technique highlighted the observation that progressive revascularization of the tissue starts 5 days after grafting [25]. Therefore, several xenograft studies have been performed to restore earlier blood supply in an attempt to improve follicular preservation by limiting ischemic damage. Surprisingly, although angiogenesis was successively promoted, better vascularization was not always associated with follicular preservation improvement [16,17,26-29]. In line with these previous publications, our results confirm the angiogenic effect of VEGF_111_. However, the impact on primordial follicular preservation was observed only in SCID mice whose xenografts were treated with VEGF_111_. Primary follicles were better preserved in NOD-SCID than in SCID mice. However, the low density of primary follicles observed in the biopsies after 3 weeks of transplantation suggests that the biological significance of these results is questionable. Moreover, heterogeneous follicular distribution within sheep ovarian cortex may have an impact on the results [21]. Follicle preservation or functional vascular network and fibrotic surface analyses did not reveal any significant differences between the two mouse strains; however, as previously described, we confirmed that ovarian tissue transplantation itself induces fibrosis [6]. Histological analysis indicates that ovarian tissue transplantation in SCID mice better preserves global tissue architecture and follicular morphology in comparison to tissue grafted in NOD-SCID mice. Other studies have also reported that damage to stromal cells as well as follicular alterations occur during the first days after transplantation [6,7]. Immunodeficient mice constitute a valuable tool for studying xenografts and tumorigenesis. Since 1960, athymic mice (nude) have been the standard for establishing *in vivo* models of human malignancies. An attractive alternative to this model is SCID mice, which lack functional T and B cells but retain normal natural killer (NK) cell activity. NOD-SCID mice have additional immunologic defects, including low NK cell function and absence of circulating complement. Currently, there is no consensus in the literature regarding the type of immunodeficient mice to use for frozen-thawed ovarian tissue xenotransplantation. Recently, different grafting sites were evaluated in nude mice; all transplantation sites equally supported follicular growth and preserved quiescent follicles and stromal fibrosis [30].

In the context of ovarian tissue recovery after autotransplantation, immunity may play an important role that is overlooked in studies using immunodeficient mice for xenografts. Indeed, to be more representative of the clinical process, the use of humanized animal models could be interesting [31]. In fact, to observe the “pure” human immune response, the recipient mice should express no mouse major histocompatibility complex (MHC) genes or express certain human leukocyte antigens (HLA).

## Conclusions

To our knowledge, this is the first study undertaken to test and compare two different immunodeficient mouse strains with a BALB/c background (SCID and NOD-SCID) as hosts and to assess the benefit of grafts treated with VEGF_111_. Our results indicate that sheep ovarian tissue xenografts in SCID and NOD-SCID mice are both suitable for studying graft recovery. However, based on histologic analysis, the overall tissue morphology is better preserved in SCID mice. Moreover, in this mouse strain, grafted ovarian tissues treated with VEGF isoform 111 display better graft perfusion and primordial follicle preservation.

## Abbreviations

SCID: Severe combined immunodeficient
NOD-SCID: Non-obese diabetic severe combined immunodeficient
NK: Natural killer
VEGF: Vascular endothelial growth factor
FITC: Fluorescein isothiocyanate
RT: Room temperature
PBS: Phosphate-buffered saline
MHC: Major histocompatibility complex
HLA: Human leukocyte antigens
